# Thermodynamic surprises of Cu(II)–amylin analogue complexes in membrane mimicking solutions

**DOI:** 10.1038/s41598-021-04197-5

**Published:** 2022-01-10

**Authors:** Emilia Dzień, Dorota Dudek, Danuta Witkowska, Magdalena Rowińska-Żyrek

**Affiliations:** 1 Faculty of Chemistry, University of Wrocław, F. Joliot-Curie 14, 50-383 Wrocław, Poland; 2 Institute of Health Sciences, University of Opole, Katowicka 68, 45-060 Opole, Poland

**Keywords:** Chemical biology, Coordination chemistry, Inorganic chemistry

## Abstract

Membrane environment often has an important effect on the structure, and therefore also on the coordination mode of biologically relevant metal ions. This is also true in the case of Cu(II) coordination to amylin analogues—rat amylin, amylin_1–19_, pramlintide and Ac-pramlintide, which offer N-terminal amine groups and/or histidine imidazoles as copper(II) anchoring sites. Complex stabilities are comparable, with the exception of the very stable Cu(II)–amylin_1–19_, which proves that the presence of the amylin C-terminus lowers its affinity for copper(II); although not directly involved, its appropriate arrangement sterically prevents early metal binding. Most interestingly, in membrane-mimicking solution, the Cu(II) affinities of amylin analogues are lower than the ones in water, probably due to the crowding effect of the membrane solution and the fact that amide coordination occurs at higher pH, which happens most likely because the α-helical structure, imposed by the membrane-mimicking solvent, prevents the amides from binding at lower pH, requiring a local unwinding of the α-helix.

## Introduction

Protein misfolding and formation of amyloidogenic aggregates are features of disorders belonging to the group of protein misfolding diseases (PMDs) including Type 2 Diabetes Mellitus (T2DM). Approximately 422 million people worldwide suffer from type 2 diabetes, which accounts for about 95% of all diabetes patients^1^. In 90% of cases, autopsy of patients with T2DM reveals the presence of amyloid deposits of amylin in the pancreas^2,3^. Amylin, also known as islet amyloid peptide (IAPP), is a 37-residue neuroendocrine peptide hormone, co-produced and co-secreted with insulin by pancreatic Langerhans β-cells^4^. Amylin has an amidated C-terminus and one intramolecular disulfide bond between Cys2 and Cys7^5^; it regulates blood glucose levels, suppresses glucagon release from the pancreas, promotes satiation and regulates gastric emptying^6,7^. Another, less known but definitely interesting property is the antimicrobial activity of amylin^8^. Under physiological conditions, human amylin (hIAPP) is present in the form of a soluble monomer, but in T2DM patients, it undergoes conformational changes**.** In the initial phase, protein monomers aggregate, resulting in the formation of α-helical fibrils, while mature forms adopt the β-sheet structure^9,10^. The most cytotoxic forms are amylin β-sheet-rich oligomers^11,12^, i.e. the pre-fibrillary structures that arise during the conversion of the native form of amylin to fibrils^13–15^.

**Figure 1:**
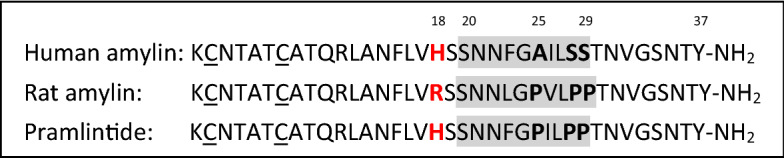
A comparison of the native sequence of human amylin, rat amylin and pramlintide. The region with the highest amyloidogenic potential is shown in gray.

To accurately understand the effect of amylin on the pathogenesis of diabetes, it is necessary to solve the mechanism of fibrillation and to know the factors that influence this process. Analysis of amino acid sequence differences between non-fibrillating rat amylin and fibrillating human amylin allowed to determine the amino acid residues with the greatest impact on fibrillation. The region with the highest amyloidogenic potential appears to be the 20–29 residues region^16^, with particular emphasis on the proline at position 25, present in the rat amylin sequence, which probably disrupts the formation of β structures characteristic for amyloid fibrils^17^. In the human amylin sequence, an alanine residue is present at position 25. Also Ser28 and Ser29 are substituted with Pro residues in non-fibrillating and non-cytotoxic rat amylin (differences shown in Fig. 1). Taking into account the influence of individual amino acid residues, a synthetic, non-fibrillating analogue, pramlintide, was developed, which is used in the USA as an antidiabetic drug under the name Symlin^®^^18^. The histidine residue at position 18 is also undoubtedly involved in the abnormal folding of amylin, as evidenced by numerous studies^16,19^. Histidine is an amino acid that is sensitive to the pH of the environment in which it is present. At acidic pH (5.5), no aggregation of amylin occurs, as observed by Khemtemourian et al.^20^ and confirmed by Li's et al. later research. They demonstrated that the protonated histidine ring prevents fibrillation by electrostatic repulsion between positively charged amylin monomers. The beginning of the fibrillation process is observed at pH 7.4 when the imidazole ring of histidine is already deprotonated^21^.

His18 is important not only for its participation in fibrillation, but is also an important binding site for zinc(II) and copper(II) ions, which are also likely involved in the pathogenesis of diabetes. Zinc(II) interactions with amylin were intensively studied due to its high concentration in pancreatic β-cells (10–20 mM)^22,23^, and because reduced levels of this metal ion are observed in T2DM patients^24^. In the case of pramlintide, and its membrane disrupting fragment, amylin_1–19_, the zinc(II) ions are coordinated by the imidazole nitrogen at position 18 and the N-terminal amino group of Lys1, leading to the bending of the peptide backbone^16,25^. However, as shown in a recent study by Khemtemourian et al., conducted in the presence of a lipid membrane using amylin analogues in which His18 was replaced by other amino acid residues, the presence of zinc(II) ions does not affect the fibrillation process of amylin^26^. It has been confirmed in Brender’s NMR experiment which showed the N-terminus is involved in interactions with the membrane and becomes inaccessible to zinc(II)^27^. This contradicts earlier reports that zinc(II) ions, depending on their concentration, may have an inhibitory^28^ or stimulatory effect on the peptide folding process^29,30^.

The role of copper(II) ions in fibril formation is also a matter of debate. On one hand, copper(II) ions limit fibril formation by increasing the activation energy in the lag phase of fibril formation, and as Sinopoli et al. showed, Cu(II)–amylin complexes do not form β structures, which are characteristic of amyloid fibrils^31^. One the other hand, inhibition of amyloid deposit formation may also be an effect of binding copper(II) ions by amylin. Coordination occurs through the N-terminal amino group and imidazole ring of His18, confirming a role for this amino acid in fibrillation^16,32,33^. The same coordination mode is observed for the Cu(II)–amylin_1–19_ complex, as confirmed by our previous work^16^. Also in the case of the copper(II) ion complex with pramlintide, one possible coordination mode is {N_im,_ 3N^−^}, next to the other mode where Cu(II) ions are coordinated by N-terminal and three amide nitrogen atoms^34^. The N- and C-terminally protected fragment of rat amylin, which lacks both the N-terminal amine group and the histidine imidazole (residues 17–29, Ac-VRSSNNLGPVLPP-NH_2_), binds Cu(II) already at pH above 6. Its even shorter region, the tetrapeptide Ac-SSNN-NH_2_, is also able to bind Cu(II)—via the deprotonated amide nitrogens, and the NH^−^ of the side chain of asparagine (as an anchoring group)^35^.

In the N-terminally free form of the same tetrapeptide, the amino terminus was the primary Cu(II) binding site, but the thermodynamic stability of the complex was additionally enhanced by the asparaginyl moiety^36^.

It is also important to keep in mind that the high redox potential of copper promotes the reduction of copper(II) ions to copper(I), which then catalyzes the Fenton reaction, leading to the formation of reactive oxygen species. Excess ROS can accelerate fibrillation and also cause β-cell damage^37^.

The mechanism of amylin cytotoxicity is based on the destruction of cell membranes by the formation of amyloid channels, which are composed of many dynamic subunits, loosely associated together to form heterogeneous channel-like structures^38^. The formation of similar structures in the membrane is also observed for many antimicrobial peptides, including protegrin-1 (PG-1). Moreover, theoretical studies show that the organization of the β subunits in the membrane channels formed by protegrin-1 and β-amyloid (Aβ) is largely identical^39^. Both the similarity of action and structure allowed to classify amylin to the group of antimicrobial peptides (AMPs). Studies of Wang et al. showed that amylin exhibits antimicrobial properties against Gram-positive *S. aureus* and Gram-negative *E. coli*. At physiological pH, amylin monomers are positively charged, which results in electrostatic attraction with the negatively charged *S. aureus* membrane. The initially disordered structure takes the shape of a helix, making it possible to anchor the peptide to the membrane. After reaching the appropriate concentration, the membrane breaks and the peptides form a micelle-like structure (the so-called carpet model). Fibrillar structures are much less toxic to membranes and are probably parallel to the bacterial cell membrane, and over time form insoluble amyloid deposits^8^.

There is no doubt that the cytotoxicity of amylin is related to its interaction with the cell membrane. As a cationic peptide, initial attraction of amylin to cell membrane is most probably driven by electrostatic interactions between positively charged amino acid side chains and negatively charged lipid membranes^40^. Engel et al. have observed that the N-terminal part of amylin, highly conserved among different species, is most probably responsible for the process of its insertion into the membrane^41^. In addition to the above-described mechanism of the formation of membrane channels, amylin in the presence of lipid membranes forms small aggregates, such as oligomers, in an aqueous solution, which further accumulate in the lipid bilayer. As aggregation progresses, some lipids are captured by newly formed aggregates which eventually cause local disruption, while the membrane acts as a matrix for further aggregation (detergent-like mode)^42^. Moreover, it has been observed that amylin, as an amyloid protein, is more prone to misfolding and forming toxic aggregates in the presence of a membrane surface^43,44^. The presence of biological membranes is known to affect the binding mode of copper(II) ions, both in terms of the type of donor atoms and affinity. Among amyloid proteins, such as human prion protein (hPrP)^45^ or chicken prion protein (chPrP)^46^, a change in secondary structure from random coil to α-helix is observed. The conformational change affects the interaction with copper(II) ions.

Because of the crucial impact of membrane environment on the structure and metal binding ability of amylin, in this work, we present the coordination chemistry of copper(II) complexes with non-amyloidogenic rat amylin, the non-aggregating fragment of amylin 1–19, pramlintide and Ac-pramlintide in membrane mimicking environment (SDS, sodium dodecyl sulfate).

## Results and discussion

Structural and thermodynamic properties of Cu(II) complexes with rat amylin, amylin_1–19_, pramlintide and Ac-pramlintide were studied and compared to each other by using mass spectrometry, potentiometry, UV–Vis and CD spectroscopy. The mass spectrometric measurements provided information about the stoichiometry of the formed complexes. The combined UV–Vis and CD results allowed to conclude the binding mode of copper(II) and the geometry of these species formed in solution, while the potentiometric titrations were the basis for the determination of precise stability constants and pH-dependent species distribution diagrams for the studied systems.

### Stoichiometry of Cu(II) binding

MS results for Cu(II)–rat amylin, Cu(II)–amylin_1–19_ and Cu(II)–pramlintide complexes, have already been discussed in our previous works, showing a 1:1 stoichiometry^16,34^. An analogous situation occurs for the Cu(II)–Ac-pramlintide complex—the MS signals correspond to the free ligand (*m/z* = 998.26, *z* = 4+) and the copper(II) complex (*m/z* = 1013.24, *z* = 4+) (Fig. S1). Other signals that occur in all spectra come from sodium, potassium and chloride adducts of ligands or of their Cu(II) complexes. The simulated isotopic patterns of copper(II) complexes are in a perfect agreement with the experimental ones.

### Protonation equilibria

In membrane mimicking SDS solution, rat amylin (KCNTATCATQRLANFLVRSSNNLGPVLPPTNVGSNTY-NH_2_) behaves as an H_3_L acid, with the deprotonating groups corresponding to the N-terminal amine group, the tyrosine side chain, and the lysine side chain with pK_a_ values of 7.85, 10.08 and 10.44, respectively. In the case of amylin_1–19_ (KCNTATCATQRLANFLVHS-NH_2_) three protonation constants were detected, corresponding to the histidine imidazole, the N-terminal amine group and the lysine side chain group, with pK_a_ values of 7.28, 8.18 and 9.88, respectively. Pramlintide (KCNTATCATQRLANFLVHSSNNFGPILPPTNVGSNTY-NH_2_) behaves as an H_4_L acid, with the deprotonating groups corresponding to the histidine imidazole, N-terminal amine group, and the tyrosine and lysine side chain groups, with pK_a_ values of 6.76, 8.39, 9.93 and 10.92, respectively. Three protonation constants were calculated for Ac-pramlintide (Ac-KCNTATCATQRLANFLVHSSNNFGPILPPTNVGSNTY-NH_2_) and were related to deprotonation of the histidine imidazole, the tyrosine side chain groups and lysine side chain group, with pK_a_ values of 7.57, 10.01 and 10.50, respectively. The cysteine groups are bridged with a disulfide bond, and the C-terminal amino acids are amidated as in the wild type forms of ligands. The comparison between protonation constants of amylin-like ligands in SDS and water solutions is shown in Table S1.

**Figure 2:**
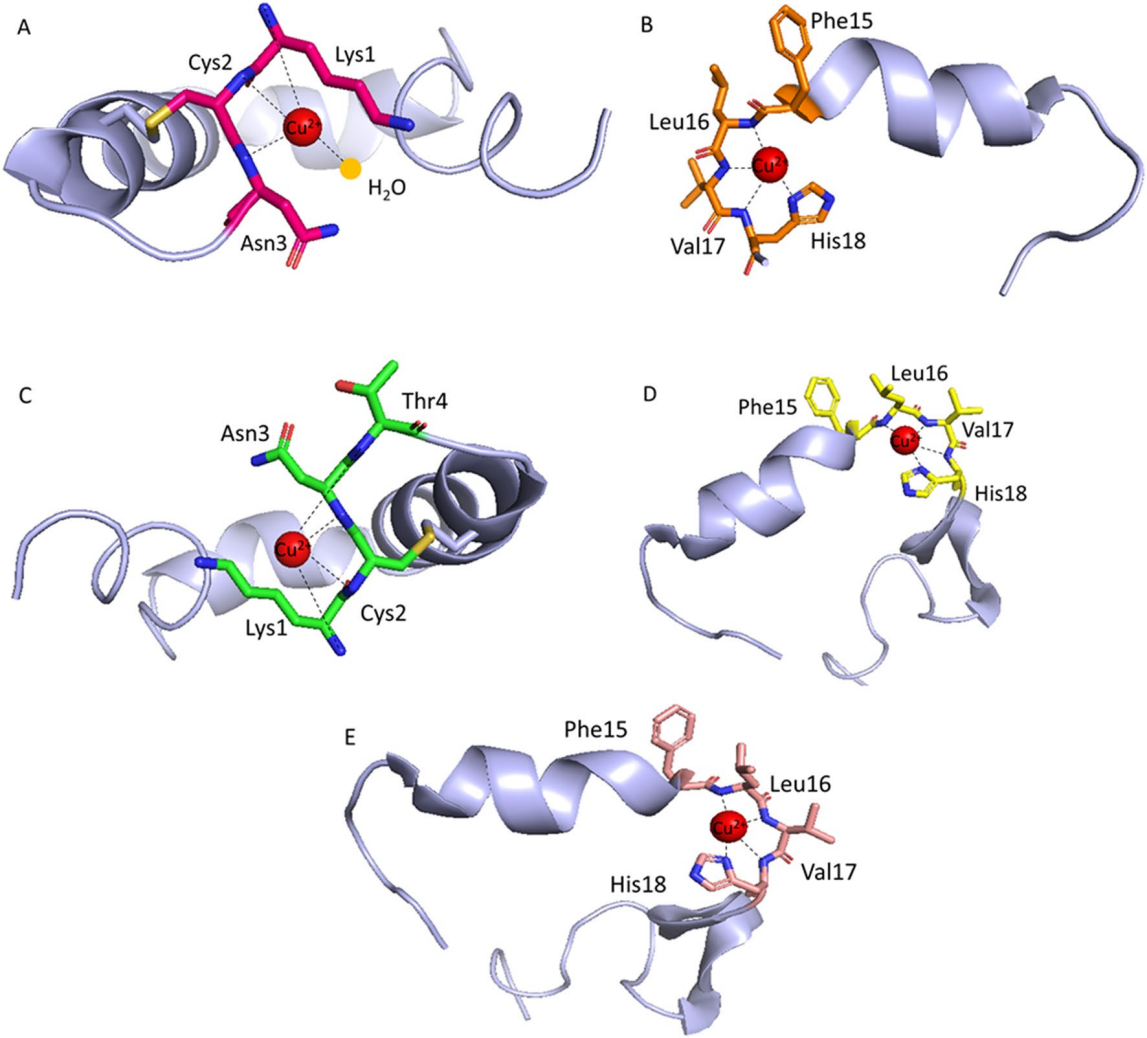
Proposed coordination mode of (**A**) Cu(II)–rat amylin complex; (**B**) Cu(II)–amylin_1–19_ complex; (**C**) Cu(II)–pramlintide complex (Cu(II) ions are coordinated by N-terminal amine and three amide nitrogen atoms) and (**D**) Cu(II)–pramlintide complex (Cu(II) ions are coordinated by the His18 imidazole and three neighboring amide nitrogen atoms) and (**E**) Cu(II)–Ac-pramlintide complex in 40 mM SDS at basic pH.

The coordination of Cu(II) to rat amylin begins at pH 4 (Fig. S2). The maximum of the [CuHL]^3+^ form occurs around pH 6.5, with the N-terminal amine group and the closest amide involved in coordination, resulting in a pronounced CT band near 290 nm and d–d transition at 581 nm in the CD spectra, respectively (Fig. S3), indicating that the complex starts to adopt a square planar geometry, with the amide nitrogen atom being involved in the coordination. The next deprotonation leads to the formation of the [CuL]^2+^ form, with a pK_a_ value of 6.83. A blue shift and an increase in intensity in CD (from 581 to 571 nm, Table S2 and Fig. S3) and UV–Vis (from 538 to 530 nm, Table S2 and Fig. S4) is observed, which confirms that one more amide is involved in coordination, resulting in an {NH_2_, 2N^−^} binding mode (Fig. 2A)^47^. The coordination sphere is completed with a water molecule. The two remaining deprotonations (pK_a_ of 9.61 and 10.04) lead to [CuH_−1_L]^+^ and [CuH_−2_L] form and are related to the deprotonation of the unbound tyrosine and lysine side chains and do not change the coordination sphere. In contrast, as our previous work showed, in water solution, the N-terminal amine group and three amide nitrogen atoms are involved in binding Cu(II) ion, resulting in an {NH_2_, 3N^−^} binding mode at pH ≈ 7.4^34^.

The first Cu(II)–amylin_1–19_ complex ([CuH_2_L]^4+^) observed at acidic pH (with a maximum above pH 5.5 (Fig. S5)), most probably involves His18 in binding. At pH 6, the maximum of the [CuHL]^3+^ complex (pK_a_ = 5.92) is observed. In the CD spectra, a CT band near 278 nm and d–d transition band at 590 nm appear (Fig. S6), and in the UV–Vis spectra, a band near 558 nm (Fig. S7) is visible. Taken together, these results suggest that His18 and an amide group are engaged in coordination at this pH. The next two deprotonations lead to the formation of [CuL]^2+^ and [CuH_−1_L]^+^ complexes, with pK_a_ values of 6.08 and 7.07, respectively. The observed shift in the CD spectra (from 590 to 580 nm and then to 574 nm, Table S2 and Fig. S6) and in UV–Vis (from 558 to 551 nm and then to 542 nm, Table S2 and Fig. S7), indicate the participation of two more amide nitrogen atoms in the metal coordination, resulting {N_im_, 3N^−^} binding mode (Fig. 2B) with a square planar geometry at pH 8. This mode does not change with further increase of pH; pK_a_ values of 8.98 and 10.05 correspond to the deprotonation of the N-terminal amine group and of the side chain of lysine, respectively; both groups do not take part in binding. In contrast, in water solution, the amide starts to participate in the binding at lower pH—the complex has a {N_im_, 3N^−^} binding mode already at pH 6^16^.

The first Cu(II)–pramlintide complex with form ([CuH_3_L]^5+^) starts form around pH 3 (Fig. S8) and reaches its maximum above pH 5.5. At this point, the His18 imidazole is most probably involved in coordination of copper(II) ions. At pH 6, the [CuH_2_L]^4+^ complex form appears (pK_a_ = 5.97) where most likely, the N-terminal amino group is involved in binding, what is confirmed by the UV–Vis band at 557 nm (Fig. S10). The loss of one proton leads to the formation of the [CuHL]^3+^ complex, with a maximum at pH 7. The CD d-d transition band shifts from 621 to 591 nm (Table S2, Fig. S9) and the UV–Vis band—from 557 to 548 nm (Table S2, Fig. S10), which suggests that an amide is directly involved in binding. Above pH 9, the [CuL]^2+^ complex dominates, with pK_a_ of 7.71. The shift and increase in intensity in the CD (from 591 to 581 nm, Table S2 and Fig. S9) and UV–Vis spectra (from 548 to 530 nm, Table S2 and Fig. S10) confirm the involvement of a second amide in the coordination. At pH 10, the next species form, [CuH_−1_L]^+^, starts to dominate in the solution, with the third amide group taking part in coordination, what is confirmed by a significant shift in the CD (from 581 to 569 nm, Table S2 and Fig. S9) and UV–Vis spectra (from 530 to 515 nm, Table S2 and Fig. S10). Most likely, at pH 10, in the Cu(II)–pramlintide complex, two forms are present in equilibrium: the first one, in which the N-terminal amine and the adjacent amides are bound to Cu(II) (Fig. 2C), and the second, where the His18 imidazole and three preceding amides are involved in the binding (Fig. 2D). The two remaining deprotonations (pK_a_ of 10.28 and 10.89), leading to [CuH_−2_L] and [CuH_−3_L]^−^ forms, are related to the deprotonation of the unbound tyrosine and lysine side chains and do not change the coordination mode. This equilibrium of the two forms is analogous to that found in water solution at pH 7.4—one with a {NH_2_, 3N^−^}, and the second, with {N_im_, 3N^−^} binding mode^34^.

The first complex form of N-terminally acetylated pramlintide, Ac-pramlintide, [CuHL]^3+^, reaches its maximum at pH 7 (Fig. S11), with the His18 imidazole and the amide group in the Cu(II) coordination sphere. The appearance of a d–d transition band at 613 nm in the CD spectra (Fig. S12) and the band near 610 nm in the UV–Vis spectra (Fig. S13), confirms that the amide nitrogen atom is involved in the coordination. The loss of next two protons leads to the formation of [CuL]^2+^ and [CuH_−1_L]^+^ complexes, with a maximum at pH 9 and 10 and pK_a_ values of 8.61 and 9.33, respectively. Shifts in the CD (from 613 to 596  nm and then to 589 nm, Table S2 and Fig. S12) and in the UV-Vis spectra (from 610 to 584 nm and then to 565 nm, Table S2 and Fig. S13) are observed, suggesting that the next two amide groups are engaged in coordination, resulting in an {N_im_, 3N^−^} coordination mode (Fig. 2E). The biding mode does not change with the increase of pH; the [CuH_−3_L]^−^ complex results from the deprotonation of the tyrosine and lysine residues, which do not take part in Cu(II) binding.

### Comparison of metal binding abilities

To compare the binding ability of Cu(II) to all investigated amylin analogues, competition plots were made, which are based on the calculated complex stability constants and show a hypothetical situation, in which equimolar amounts of the compared ligands and Cu(II) are present. Up to pH 5, the abundance of Cu(II)–amylin_1–19_ and Cu(II)–pramlintide complexes are nearly identical. At this point, both ligands coordinate Cu(II) via one imidazole group. At higher pH values, amylin_1–19_ binds Cu(II) via His18 and three amide nitrogen atoms, while the pramlintide Cu(II) complex is most likely observed the presence of two forms in equilibrium—one with a {NH_2_, 3N^−^}, and the second, with {N_im_, 3N^−^} binding mode.

Above pH 6, Cu(II)–amylin_1–19_ becomes the most thermodynamically stable among all analyzed complexes, suggesting that the presence of the C-terminal part of amylin analogues lowers their affinity towards copper(II). The C-terminus plays an auxiliary, yet crucial, role in binding; its appropriate arrangement makes metal binding sterically unfavorable and prevents early binding of Cu(II), thus improving thermodynamic stability of the complex, even if it is not directly involved.

**Figure 3:**
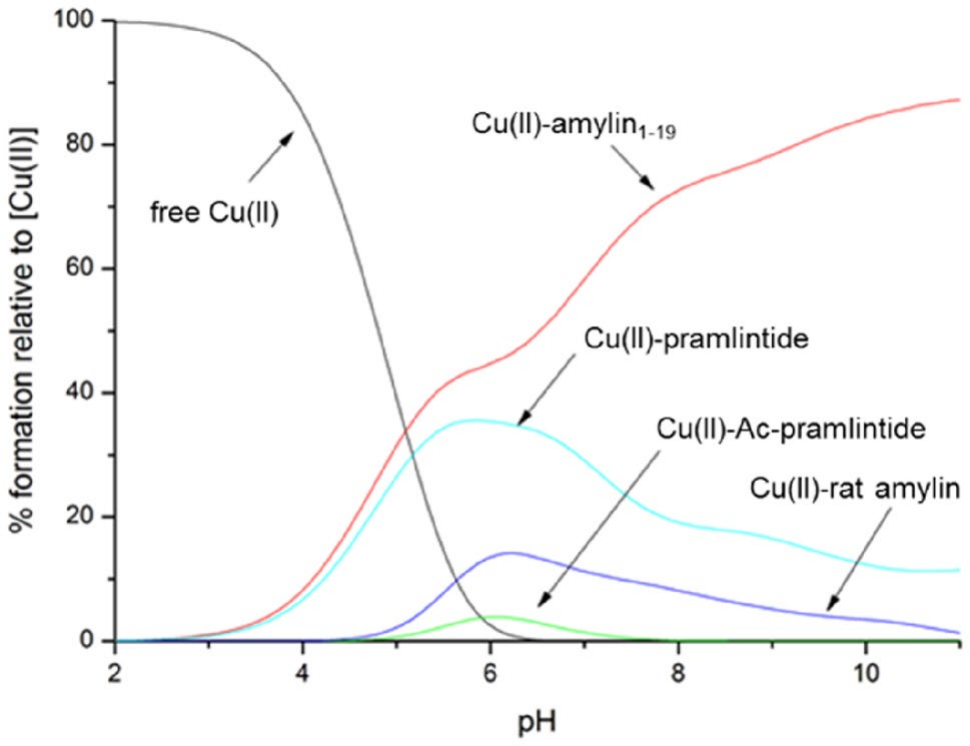
Competition plot between Cu(II) complexes of rat amylin, amylin_1–19_, pramlintide and Ac-pramlintide in 40 mM SDS solution. Describes complex formation at different pH values in a hypothetical situation in which equimolar amounts of the five reagents are mixed. Calculations are based on binding constants from Table S2. Conditions: T = 298 K, [Cu(II)] = [rat amylin] = [amylin_1–19_] = [pramlintide] = [Ac-pramlintide] = 0.001 M.

The stability of the N-terminally bound Cu(II) complex with rat amylin is lower than that of amylin_1–19_ and pramlintide, but slightly higher than that of Cu(II)–Ac-pramlintide, which includes imidazole and amide nitrogen atoms in the coordination sphere, which points out the relevance of the presence of the free N-terminus in the discussed ligands (Fig. 3).

**Figure 4:**
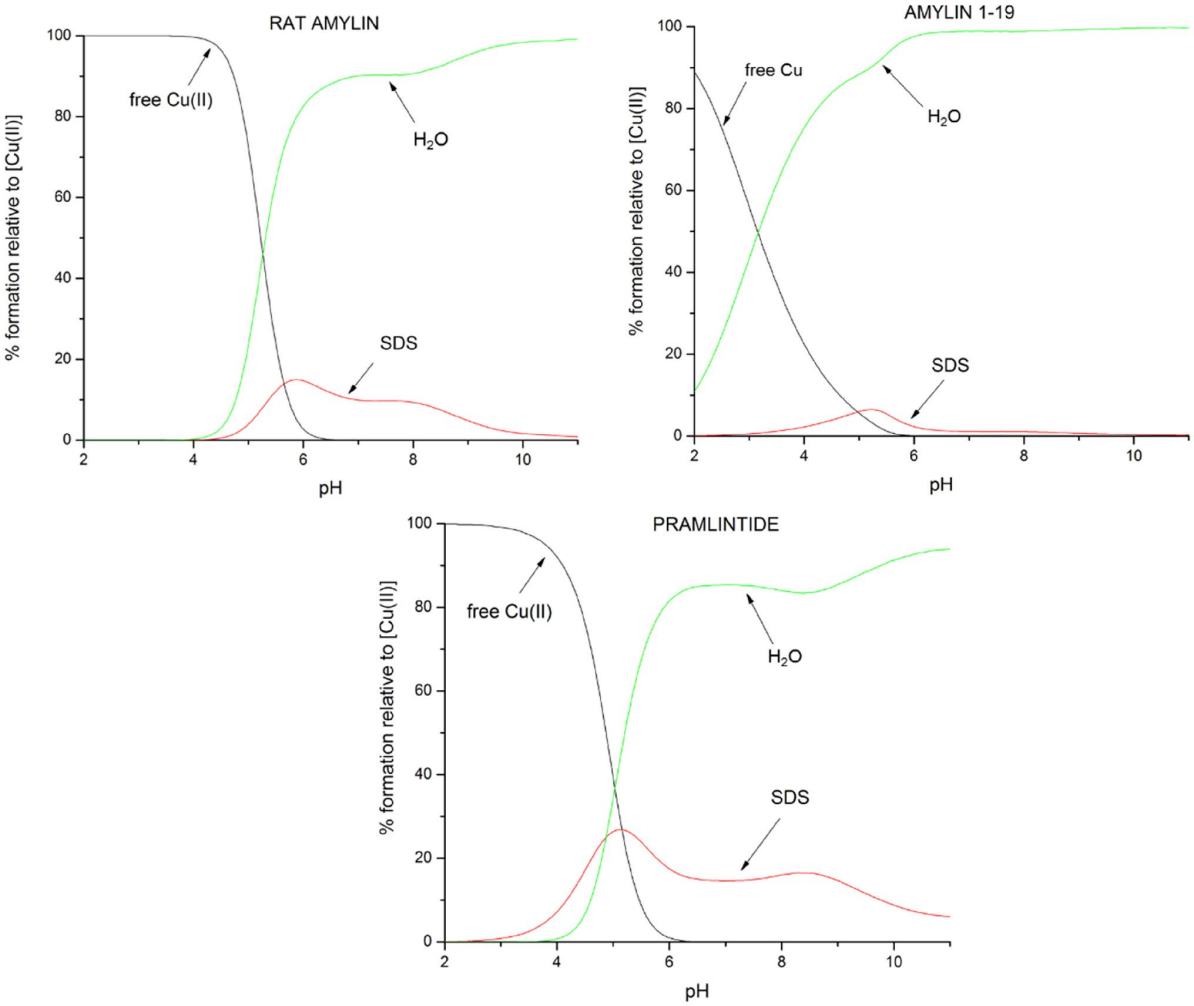
Competition plots between Cu(II) complexes of rat amylin, amylin_1–19_ and pramlintide in both water and 40 mM SDS solutions. Describes complex formation at different pH values in a hypothetical situation in which equimolar amounts of the three reagents are mixed. Calculations are based on binding constants from previously published data^16,34^. Conditions: T = 298 K, [Cu(II)] = [rat amylin] = [amylin_1–19_] = [pramlintide] = 0.001 M.

The binding modes (Tables 1 and 2) and affinities (Fig. 4) of the studied ligands in 40 mM SDS presented in this work were compared to those in water solution (data for amylin_1–19_ taken from ref. 16 and for rat amylin and pramlintide from Ref.^34^). Thermodynamic stability of all presented complexes is significantly higher in water solutions than in SDS. Also, in all cases, square planar geometry of complexes starts to form at a lower pH value (pH ≈ 6) for water solutions when compared to SDS solutions (pH ≈ 7). In in the presence of membrane-mimicking solution, all studied amylin analogues and their Cu(II) complexes adopt helical-like structures, showing two characteristic minima at 222 and 208 nm and one maximum at 193 nm in the CD spectra (Fig. S14). This suggests that (1) SDS may act as a crowding agent and that (2) the α-helical structure, imposed by the solvent, ‘protects’ the amides from binding—the structure has to unwind, at least locally, in order to form a square planar complex with Cu(II) ions—a similar effect was observed in α-helical, His-rich peptides^48–50^.

**Table 1:** Summary of proposed binding modes in both 40 mM SDS and water solution for investigated amylin analogues at pH = 7.4.

Ligand	40 mM SDS	H_2_O^16,34^
Rat amylin	NH_2_, 1N^−^/2N^−^	N_im_ 2N^−^
Amylin_1–19_	N_im_, 2N^−^	N_im_, 3N^−^
Pramlintide	N_im_, NH_2,_ 1N^−^	N_im_, 3N^−^ or NH_2_, 3N^−^
Ac-pramlintide	N_im_, 1N^−^	–

**Table 2:** Summary of proposed binding modes in both 40 mM SDS and water solution for investigated amylin analogues at pH = 10.

Ligand	40 mM SDS	H_2_O^16,34^
Rat amylin	NH_2_, 2N^−^	N_im_ 3N^−^
Amylin_1–19_	N_im_, 3N^−^	N_im_, 3N^−^
Pramlintide	N_im_, 3N^−^ or NH_2_, 3N^−^	N_im_, 3N^−^ or NH_2_, 3N^−^
Ac-pramlintide	N_im_, 3N^−^	–

## Conclusion

The work summarizes details of Cu(II) coordination to four amylin analogues (rat amylin, amylin_1–19_, pramlintide and Ac-pramlintide) in a biological membrane mimicking environment. Rat amylin coordinates Cu(II) ions through the N-terminal amino group and two amide nitrogen atoms (Fig. 2A). Binding of Cu(II) ions to amylin_1–19_ and Ac-pramlintide occurs through the histidine imidazole ring in position 18 and three nitrogen atoms from the amide group of the peptide bond which precede His18 (Fig. 2B,E). In the case of the Cu(II)–pramlintide complex, at basic pH, two species probably coexist together in solution: (1) one in which the N-terminus of the polypeptide and three neighboring amides are involved in the coordination of Cu(II) (Fig. 2C) and (2) another one, in which the His18 and three neighboring amides bind the metal ion (Fig. 2D). The findings for amylin_1–19_, rat amylin and pramlintide copper(II) complexes are in good agreement with the ones found in water environment, with two significant differences—in SDS membrane-mimicking solution, the affinities of amylin analogues are lower than the ones in water (probable crowding effect of membrane solution), and amide coordination occurs at higher pH, which is most likely due to the fact that the α-helical structure, imposed by the membrane-mimicking solvent, ‘protects’ the amides from binding at lower pH.

Our findings confirm the importance of His18 in the coordination of Cu(II) ions, but most importantly, they make us realize the effect of membrane mimicking environment on the structure, and therefore also the coordination mode of amylin analogues.

## Methods

### Synthesis

All the ligands, the C-protected disulfide-bridged rat amylin (KCNTATCATQRLANFLVRSSNNLGPVLPPTNVGSNTY-NH_2_), C-protected disulfide bridged amylin_1–19_ (KCNTATCATQRLANFLVHS-NH_2_), C-protected disulfide bridged pramlintide (KCNTATCATQRLANFLVHSSNNFGPILPPTNVGSNTY-NH_2_) and C-protected disulfide bridged Ac-pramlintide (Ac-KCNTATCATQRLANFLVHSSNNFGPILPPTNVGSNTY-NH_2_) were purchased from KareBayBiochem (USA) (certified purity of 99.30%) and used as received. The purity was checked potentiometrically. The Cu(ClO_4_)_2_ was an extra pure product (Sigma-Aldrich). The carbonate-free stock solution of 0.1 M NaOH was purchased from Merck and then potentiometrically standardized with potassium hydrogen phthalate.

### Mass spectrometry

High-resolution mass spectra was obtained on a BruckerQ-FTMS spectrometer (Bruker Daltonik, Bremen, Germany), equipped with Apollo II electrospray ionization source with an ion funnel. The mass spectrometer was operated both in the positive ion mode. The instrumental parameters were as follows: scan range m/z 300–3000, dry gas—nitrogen, temperature 170 °C, ion energy 5 eV. Capillary voltage was optimized to the highest S/N ratio and it was 4500 V. The small changes of voltage (± 500 V) did not significantly affect the optimized spectra. The samples (Cu(II):ligand in a 0.9:1 stoichiometry, [ligand]_tot_ = 10^−4^ M) were prepared in 1:1 acetonitrile-water mixture at pH 7.4. The variation of the solvent composition down to 5% of acetonitrile did not change the species composition. The sample was infused at a flow rate of 3 μL/min. The instrument was calibrated externally with the Tunemix™ mixture (BrukerDaltonik, Germany) in the quadratic regression mode. Data were processed by using the Bruker Compass DataAnalysis 4.0 program. The mass accuracy for the calibration was better than 5 ppm, enabled together with the true isotopic pattern (using SigmaFit) an unambiguous confirmation of the elemental composition of the obtained complex.

### Potentiometric measurements

Stability constants for proton and Cu(II) complexes were calculated from titration curves carried out over the pH range 2–11 at 298 K and 40 mM SDS ionic strength using a total volume of 3 cm^3^. The potentiometric titrations were performed using a Dosimat 665 Metrohm titrator connected to a Metrohm 691 pH-meter and a Mettler Toledo, InLab microglass electrode. The thermostabilized glass-cell was equipped with a magnetic stirring system, a microburet delivery tube and an inlet-outlet tube for argon. Solutions were titrated with 0.1 M carbonate-free NaOH. The electrodes were daily calibrated for hydrogen ion concentration by titrating HClO_4_ with NaOH in the same experimental conditions as above. The purities and the exact concentrations of the ligand solutions were determined by the Gran method^51^. The ligand concentration was 0.5 mM, the Cu(II) to ligand ratio was 0.9:1. HYPERQUAD 2006 program was used for the stability constant calculations^52^. Standard deviations were computed by HYPERQUAD 2006 and refer to random errors only. The speciation and competition diagrams were computed with the HYSS program^53^.

### Spectroscopic studies

Solutions were of similar concentrations with respect to those used in the potentiometric studies. Absorption spectra were recorded on a Cary 300 Bio spectrophotometer, in the range of 200–800 nm, using a quartz cuvette with an optical path of 1 cm. Circular dichroism (CD) spectra were recorded on a Jasco J 715 spectropolarimeter in the 240–800 nm range, using a quartz cuvette with an optical path of 1 cm in the visible and near-UV range and 0.01 cm in the spectral range of 180–260 nm. The UV–Vis and CD spectroscopy parameters were calculated from the spectra obtained at the pH values corresponding to the maximum concentration of each particular species, on the basis of potentiometric studies.

## Supplementary Information

Supplementary Information.
