# Supplementary material for: Thermodynamic surprises of Cu(II)–amylin analogue complexes in membrane mimicking solutions
Source: Sci Rep. 2022 Jan 10;12:425. doi: 10.1038/s41598-021-04197-5 (PMC8748748; doi:10.1038/s41598-021-04197-5)
Supplement: Supplementary file 1 — Supplementary Information. [file 41598_2021_4197_MOESM1_ESM.docx]

**Supplementary information**

**Thermodynamic surprises of Cu(II)-amylin analogue complexes in membrane mimicking solution**

# Emilia Dzień^1^, Dorota Dudek^1^, Danuta Witkowska^2,*^, and Magdalena Rowińska-Żyrek^1,*^

^1^Faculty of Chemistry, University of Wroclaw, F. Joliot-Curie 14, 50-383 Wroclaw, Poland

^2^Institute of Health Sciences, University of Opole, Katowicka 68, 45-060 Poland

^*^magdalena.rowinska-zyrek@chem.uni.wroc.pl

^*^danuta.witkowska@uni.opole.pl

**
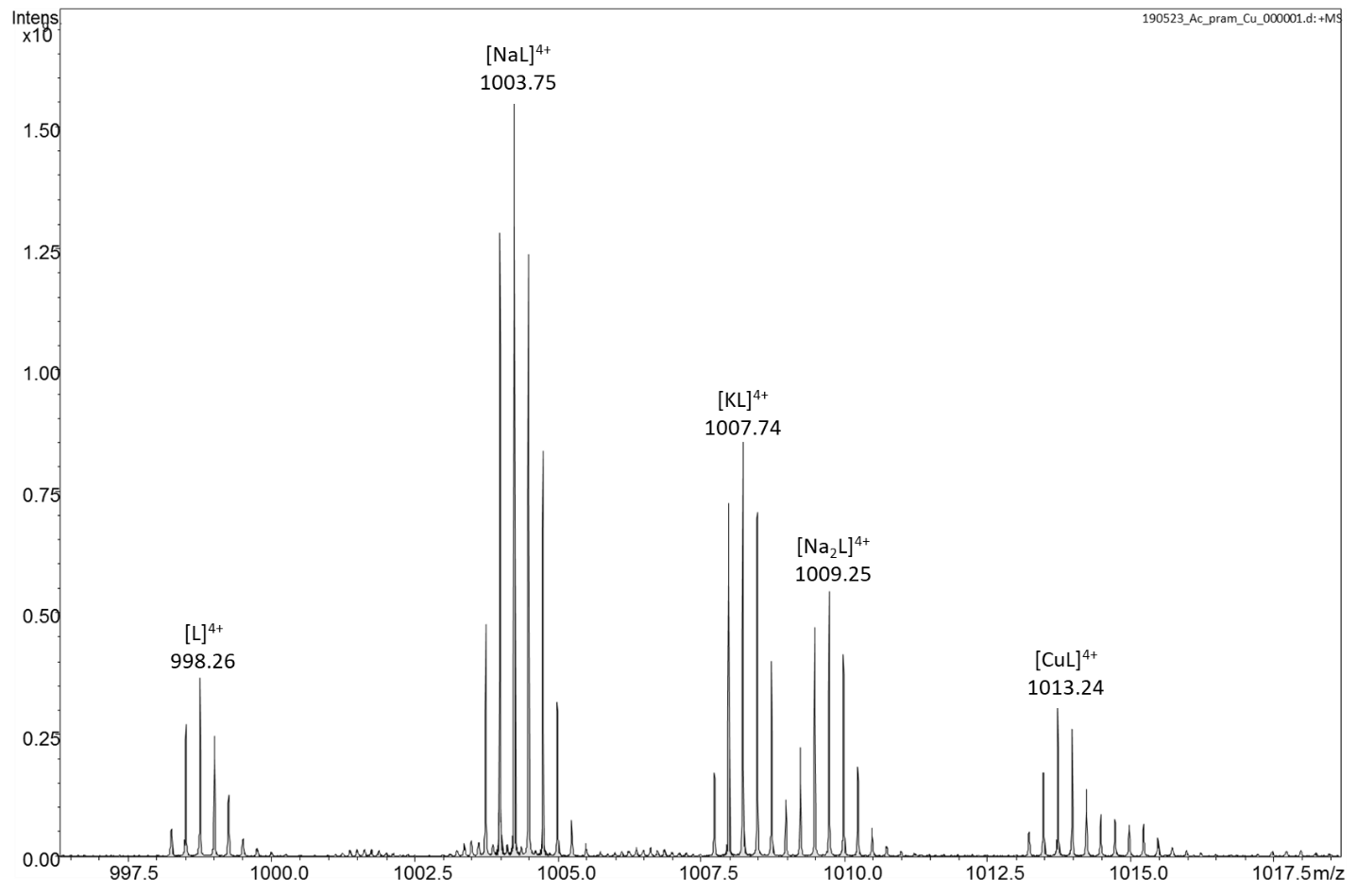
**

**Figure S1**. ESI-MS spectrum of Cu(II)-Ac-pramlintide at pH 7.4. M:L molar ratio = 1:1.

**Table S1.** Protonation constants of rat amylin, amylin_1-19_, and pramlintide in 40 mM SDS solution and water solution.

|  | SDS | | H_2_O^1,2^ | |
| --- | --- | --- | --- | --- |
| species | logβ | pK_a_ | logβ | pK_a_ |
| **rat amylin** | | | | |
| [HL]^+^ | 10.44(4) | 10.44 (K) | 10.20(4) | 10.20 (K) |
| [H_2_L]^2+^ | 20.51(3) | 10.08 (Y) | 19.69(3) | 9.49 (Y) |
| [H_3_L]^3+^ | 28.39(9) | 7.85 (N-t) | 26.82(6) | 7.13 (N-t) |
| **amylin_1-19_** | | | | |
| [HL]^+^ | 9.88(2) | 9.88 (K) | 10.83 | 10.83 (K) |
| [H_2_L]^2+^ | 18.06(4) | 8.18 (N-t) | 18.74 | 7.97 (N-t) |
| [H_3_L]^3+^ | 25.34(4) | 7.28 (H) | 24.78 | 6.04 (H) |
| **pramlintide** | | | | |
| [HL]^+^ | 10.92(1) | 10.92 (K) | 10.32(1) | 10.32 (K) |
| [H_2_L]^2+^ | 20.85(1) | 9.93 (Y) | 19.98(1) | 9.67 (Y) |
| [H_3_L]^3+^ | 29.24(2) | 8.39 (N-t) | 27.51(1) | 7.53 (N-t) |
| [H_4_L]^4+^ | 36.0(3) | 6.76 (H) | 33.31(1) | 5.79 (H) |
| **Ac-pramlintide** | | | | |
| [HL]^+^ | 10.50 | 10.50 (K) | - | - |
| [H_2_L]^2+^ | 20.51 | 10.01 (Y) | - | - |
| [H_3_L]^3+^ | 28.08 | 7.58 (H) | - | - |

**Table S2.** Potentiometric and spectroscopic data for Cu(II) complexes with rat amylin, amylin_1-19_, pramlintide and Ac-pramlintide in 40 mM SDS solution.

|  | potentiometry | | UV-Vis | | CD | |
| --- | --- | --- | --- | --- | --- | --- |
| species | logβ | pK_a_ | λ [nm] | ε [M^-1^cm^-1^] | λ [nm] | Δε [M^-1^cm^-1^] |
| **rat amylin** | | | | | | |
| ligand |  |  |  |  |  |  |
| [HL]^+^ | 10.44(4) | 10.44 (K) |  |  |  |  |
| [H_2_L]^2+^ | 20.51(3) | 10.08 (Y) |  |  |  |  |
| [H_3_L]^3+^ | 28.39(9) | 7.85 (N-t) |  |  |  |  |
| **Cu(II) complex** |  |  |  |  |  |  |
| [CuHL]^3+^ | 20.11(4) |  | 538 | 117.56 | 581.6  480.2  282.8 | -0.11  0.05  -0.20 |
| [CuL]^2+^ | 13.28(4) | 6.83 | 530 | 159.96 | 571.5  288.1 | -0.18  -0.19 |
| [CuH_-1_L]^+^ | 3.67(6) | 9.61 | 519 | 216.03 | 568.1  478.0  320.9 | -0.26  0.08  0.13 |
| [CuH_-2_L] | -6.37(5) | 10.04 |  |  | 565.0  474.2  255.2 | -0.29  0.06  -0.13 |
| **amylin_1-19_** | | | | | | |
| ligand |  |  |  |  |  |  |
| [HL]^+^ | 9.88(2) | 9.88 (K) |  |  |  |  |
| [H_2_L]^2+^ | 18.06(4) | 8.18 (N-t) |  |  |  |  |
| [H_3_L]^3+^ | 25.34(4) | 7.28 (H) |  |  |  |  |
| **Cu(II) complex** |  |  |  |  |  |  |
| [CuH_2_L]^4+^ | 23.36(4) |  | minor | minor | minor | minor |
| [CuHL]^3+^ | 17.44(2) | 5.92 | 558 | 63.67 | 590.0  474.0  323.1  278.7  256.2 | -0.28  0.06  0.23  -0.52  0.07 |
| [CuL]^2+^ | 11.36(2) | 6.08 | 551 | 100.40 | 580.2  491.4  318.4  285.6  253.5 | -0.39  0.14  0.33  -0.51  0.63 |
| [CuH_-1_L]^+^ | 4.29(3) | 7.07 | 542 | 135.26 | 574.2  492.7  319.3  282.7  251.1 | -0.45  0.21  0.42  -0.45  1.03 |
| [CuH_-2_L] | -4.69(3) | 8.98 | 525 | 167.72 | 561.6  492.1  319.5  285.0  252.8 | -0.50  0.19  0.41  -0.55  1.12 |
| [CuH_-3_L]^-^ | -14.74(4) | 10.05 | 501 | 200.74 | 555.6  478.4  315.6  279.0  249.9 | -0.73  0.13  0.42  -1.05  0.66 |
| **pramlintide** | | | | | | |
| ligand |  |  |  |  |  |  |
| [HL]^+^ | 10.92(1) | 10.92 (K) |  |  |  |  |
| [H_2_L]^2+^ | 20.85(1) | 9.93 (Y) |  |  |  |  |
| [H_3_L]^3+^ | 29.24(2) | 8.39 (N-t) |  |  |  |  |
| [H_4_L]^4+^ | 36.0(3) | 6.76 (H) |  |  |  |  |
| **Cu(II) complex** |  |  |  |  |  |  |
| [CuH_3_L]^5+^ | 33.92(8) |  | 563 | 38.73 | minor | minor |
| [CuH_2_L]^4+^ | 27.95(5) | 5.97 | 557 | 70.01 | 624.9  314.6  273.4 | -0.11  0.37  -0.43 |
| [CuHL]^3+^ | 21.93(4) | 6.02 | 548 | 109.68 | 591.5  320.1  281.9 | -0.21  0.35  -0.24 |
| [CuL]^2+^ | 14.22(5) | 7.71 | 530 | 160.02 | 581.8  481.5  310.4  281.5 | -0.32  0.08  0.47  -0.03 |
| [CuH_-1_L]^+^ | 4.72(5) | 9.50 | 515 | 192.64 | 569.3  306.6  273.0 | -0.42  0.54  -0.18 |
| [CuH_-2_L] | -5.56(5) | 10.28 | 511 | 204.64 | 565.8  309.0  275.3 | -0.45  0.46  -0.33 |
| [CuH_-3_L]^-^ | -16.45(5) | 10.89 | - | - | - | - |
| **Ac-pramlintide** | | | | | | |
| ligand |  |  |  |  |  |  |
| [HL]^+^ | 10.50 | 10.50 (K) |  |  |  |  |
| [H_2_L]^2+^ | 20.51 | 10.01 (Y) |  |  |  |  |
| [H_3_L]^3+^ | 28.08 | 7.57 (H) |  |  |  |  |
| **Cu(II) complex** |  |  |  |  |  |  |
| [CuHL]^3+^ | 19.31(3) |  | 610 | 62.76 | 613.1  257.3 | -0.19  0.88 |
| [CuL]^2+^ | 10.70(9) | 8.61 | 584 | 82.19 | 596.0  255.5 | -0.32  1.83 |
| [CuH_-1_L]^+^ | 1.37(7) | 9.33 | 565 | 107.96 | 589.0  254.7 | -0.43  2.26 |
| [CuH_-2_L] | - | - | - | - | - | - |
| [CuH_-3_L]^-^ | -19.02(7) | - | 557 | 128.76 | 590.3  312.5  256.4 | -0.55  -0.07  2.17 |

**Figure S2**. Distribution diagrams for the formation of Cu(II) complex with rat amylin. Conditions: T = 298 K,
I = 40 mM SDS, [Cu(II)] = 0.45x10^-3^ M; M:L molar ratio = 0.9:1.

**Figure S3**. CD spectra of Cu(II)-rat amylin complex recorded at different pH values. Conditions: T = 298 K,
I = 40 mM SDS, [Cu(II)] = 0,45x10^-3^ M; M:L molar ratio = 0.9:1.

**Figure S4**. UV-Vis spectra of Cu(II)-rat amylin complex recorded at different pH values. Conditions: T = 298 K,
I = 40 mM SDS, [Cu(II)] = 0,45x10^-3^ M; M:L molar ratio = 0.9:1.

**Figure S5**. Distribution diagrams for the formation of Cu(II) complex with amylin_1-19_. Conditions: T = 298 K,
I = 40 mM SDS, [Cu(II)] = 0.45x10^-3^ M; M:L molar ratio = 0.9:1.

**Figure S6**. CD spectra of Cu(II)-amylin_1-19_ complex recorded at different pH values. Conditions: T = 298 K,
I = 40 mM SDS, [Cu(II)] = 0,45x10^-3^ M; M:L molar ratio = 0.9:1.

**Figure S7**. UV-Vis spectra of Cu(II)-amylin_1-19_ complex recorded at different pH values. Conditions: T = 298 K,
I = 40 mM SDS, [Cu(II)] = 0,45x10^-3^ M; M:L molar ratio = 0.9:1.

**Figure S8**. Distribution diagrams for the formation of Cu(II) complex with pramlintide. Conditions: T = 298 K,
I = 40 mM SDS, [Cu(II)] = 0.45x10^-3^ M; M:L molar ratio = 0.9:1.

**Figure S9**. CD spectra of Cu(II)-pramlintide complex recorded at different pH values. Conditions: T = 298 K,
I = 40 mM SDS, [Cu(II)] = 0,45x10^-3^ M; M:L molar ratio = 0.9:1.

**Figure S10**. UV-Vis spectra of Cu(II)-pramlintide complex recorded at different pH values. Conditions: T = 298 K,
I = 40 mM SDS, [Cu(II)] = 0,45x10^-3^ M; M:L molar ratio = 0.9:1.

**Figure S11**. Distribution diagrams for the formation of Cu(II) complex with Ac-pramlintide. Conditions:
T = 298 K, I = 40 mM SDS, [Cu(II)] = 0.45x10^-3^ M; M:L molar ratio = 0.9:1.

**Figure S12**. CD spectra of Cu(II)-Ac-pramlintide complex recorded at different pH values. Conditions: T = 298 K,
I = 40 mM SDS, [Cu(II)] = 0,45x10^-3^ M; M:L molar ratio = 0.9:1.

**Figure S13.** UV-Vis spectra of Cu(II)-Ac-pramlintide complex recorded at different pH values. Conditions:
T = 298 K, I = 40 mM SDS, [Cu(II)] = 0,45x10^-3^ M; M:L molar ratio = 0.9:1.

A

B

C

D

**Figure S14**. Comparison of CD spectra of (A)rat amylin and its Cu(II) complex, (B) amylin_1-19_ and its Cu(II) complex, (C) pramlintide and its Cu(II) complex and (D) Ac-pramlintide and its Cu(II) complex at pH = 7.4. Conditions: T = 298 K, I = 40 mM SDS, [Cu(II)] = 0,45x10^-3^ M; M:L molar ratio = 0.9:1, optical path = 0.01 cm.

**References:**

1. Rowinska-Zyrek, M. Coordination of Zn^2+^ and Cu^2+^ to the membrane disrupting fragment of amylin. *Dalton Trans.* **45**, 8099-8106 (2016).
2. Dudek, D. *et al*. Copper(II) And Amylin Analogues: A Complicated Relationship. *Inorg. Chem.* **59**, 4, 2527–2535 (2020).
